# Outcomes of a “virtual think tank” to establish collaborative leadership initiative plans (“CLIPs”)

**DOI:** 10.1016/j.rcsop.2024.100409

**Published:** 2024-01-18

**Authors:** Whitney D. Maxwell, Kerry K. Fierke, Gregory M. Zumach

**Affiliations:** a Department of Clinical Pharmacy and Outcomes Sciences, University of South Carolina College of Pharmacy, 715 Sumter Street, Columbia, SC 29208, United States of America; b Department of Pharmacy Practice and Pharmaceutical Sciences, University of Minnesota, College of Pharmacy, 232 Life Science, 1110 Kirby Drive, Duluth, MN 55812-3003, United States of America; c Department of Pharmacy Practice and Science, University of Nebraska Medical Center College of Pharmacy, 986120 Nebraska Medical CenterOmaha, NE 68198-6120, United States of America

**Keywords:** Leadership, Connection, Networking, Accessibility, Innovation

## Abstract

**Goal:**

The American Association of Colleges of Pharmacy (AACP) Leadership Development Special Interest Group (LD SIG) held a one-hour “Virtual Think Tank” (VTT) interactive session in 2020 for pharmacy educators interested in leadership development. The purpose of this study was to evaluate the quantitative and qualitative outcomes of this VTT.

**Methods:**

VTT attendees worked together in small groups created based on pre-selected common interest areas related to leadership development to create collaborative leadership initiative plans (CLIPs), which were ideas for new collaborative scholarly or programmatic initiatives.

**Principal findings:**

Quantitative outcomes of this VTT included statistically significant increases in positive perceptions toward the organization hosting the VTT regarding networking, scholarly collaboration, educational collaboration, and professional service opportunities, as well as significant improvements in attitudes regarding engagement with the sponsoring organization. Additionally, 18.4% of VTT attendees continued communicating with CLIP groups post-VTT and 13.2% of respondents indicated that they successfully implemented the CLIP ideas that were generated during the VTT. Qualitative outcomes included findings that the two most commonly encountered barriers were insufficient traction of the initial idea and lack of time (41.9% (*n* = 13) for both). Other barriers included lack of alignment with priorities at 12.9% (*n* = 4).

**Practical applications:**

This leadership VTT for pharmacy academicians led to development and implementation of important scholarly and programmatic outcomes, and fostered cross-institutional partnerships. Findings from this study evaluating a VTT provide a framework of expectations for other organizations seeking to implement a similar initiative.

## 1 Introduction

The virtual collaboration landscape is rich with challenges and opportunities. Virtual collaboration involves working together in a virtual setting to apply information for problem-solving.^1^^,^^2^ Virtual Think Tanks (VTT) offer a promising approach for promoting collaboration, innovation, and knowledge exchange among professionals in various fields. This article reports the findings of a study investigating the impact of a virtual think tank on leadership innovations, scholarly and programmatic initiative collaboration, and knowledge exchange among pharmacy educators.

According to an article published by Giancarlo Crocetti in 2015, Think Tanks have become a mechanism for complex problem-solving through interdisciplinary, expert collaboration. The conversations occurring during think tanks bring together a variety of perspectives and disciplines to collectively fill in knowledge gaps and draw new connections, leading to acquisition of new knowledge and novel solutions to previously unsolvable problems.^3^ Within the realm of health care, the goals identified for think tanks typically include fostering multidisciplinary collaborations, identifying priority research areas, and rapidly innovating to advance specific aspects of knowledge or care.^4^^,^^5^ During the start of the COVID-19 pandemic in the summer of 2020, virtual interaction became a necessity for pharmacy educators interested in collaborating to advance leadership development. Thus, the Authors transformed a live Think Tank, a format of small groups collaborating to identify direction and outcomes for scholarly and academic work, into a VTT. While the collaborative needs of a Think Tank are similar to a VTT, the VTT provides a flexible mechanism for collaboration in a virtual facilitated networking forum.

Despite utilization of Think Tanks in medicine and other fields, including increasing utilization of Virtual Think Tanks, much remains to be learned regarding the outcomes of VTTs.^6^, ^7^, ^8^, ^9^ The purpose of this study was to evaluate quantitative and qualitative outcomes of a VTT opportunity for pharmacy educators interested in leadership development. These outcomes include quantitative impacts of a VTT on attendees' perceptions regarding networking, scholarly collaboration, educational collaboration, professional service, and engagement with the organization providing the VTT. Additionally, this study sought to identify barriers to implementation of novel ideas generated during a VTT as well as reasons that new collaborators may not remain in communication with one another to implement novel ideas following a VTT. The professional demographics of VTT attendees and the outcomes described above are described in this study; thus, we also propose an optimal attendee profile based on our findings.

## 2 Methods

The American Association of Colleges of Pharmacy (AACP) Leadership Development Special Interest Group (LD SIG) held a one-hour “Virtual Think Tank” (VTT) interactive session during the 2020 AACP Virtual Annual Meeting. The AACP Annual Meeting is a professional meeting attended predominantly by administrators, educators, and researchers from colleges of pharmacy delivering doctoral pharmacy graduate education, most of whom are also practicing pharmacists. The broad purpose of the VTT was to connect attendees with colleagues from other institutions around the nation who share similar interests within the realm of leadership development to ideate collaboratively, identify arenas for innovation and research advancement, and ideally accelerate leadership development initiatives within pharmacy education. The specific intent was for each VTT attendee to work with a small group of new collaborators to generate an idea or Collaborative Leadership Initiative Plan (CLIP) for a programmatic or scholarly leadership development initiative and develop a preliminary plan for follow-up after the VTT to facilitate future implementation. Each CLIP group had individuals from a variety of institutions with shared leadership development related interest areas based on information gathered through email communication prior to the VTT. A Virtual Structured Introduction Form (VSIF) was emailed to individuals who submitted a RSVP to attend the VTT session. Attendees were asked to choose from six primary academic leadership interest areas on the VSIF form. These leadership interest areas align well with the pillars of academia including research, service, and teaching, which all pharmacy faculty are actively engaged in and on which their performance is evaluated at most institutions. They were also asked to identify current institutional leadership needs and existing institutional strengths/resources related to leadership development on the VSIF. The primary determinant of each CLIP group's constituency was shared primary leadership interest area, but the institutional needs and strengths were also shared digitally with VTT attendees to assist with idea generation during the VTT.

**Fig. 1:**
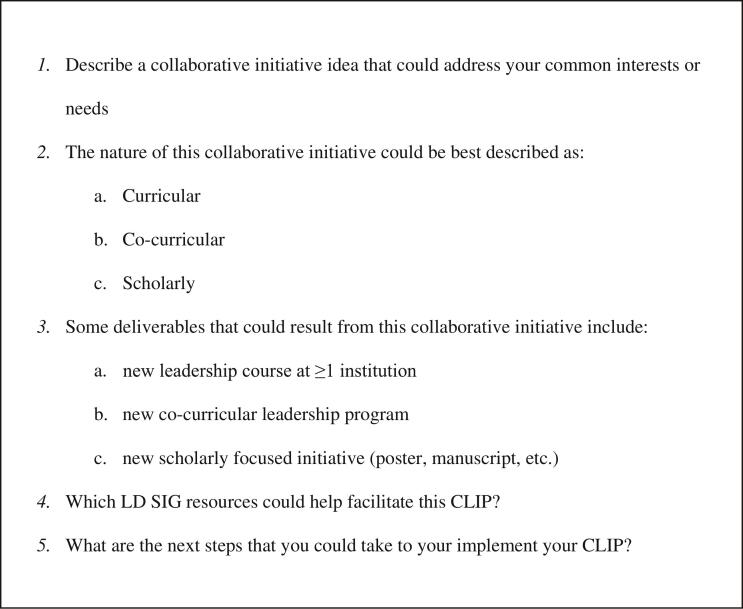
CLIP Questionnaire.

During the VTT, CLIP group facilitators used a guide to walk attendees through the VTT, enabling approximately 10 individuals per group to brainstorm and arrive at a CLIP idea to implement together. Each CLIP group also identified specific deliverables or outcomes that could result from successful implementation of their idea. Facilitators documented each group's CLIP idea(s) and anticipated outcomes on a digital worksheet called the CLIP Questionnaire (Fig. 1). Facilitators also pointed the CLIP groups to resources available from the LD SIG to assist with implementing their CLIP ideas, including but not limited to, a Leadership Toolkit and a Leadership Book Blog. The CLIP groups also discussed plans for future follow-up and shared contact information with one another.^10^

One month after the session, the VTT coordinator sent a follow-up communication to attendees to encourage continued collaboration and implementation of CLIPs. Six months post-session, attendees received a survey requesting information about CLIP group communications and CLIP outcomes such as: implementation of programmatic changes, development of scholarly outcomes, and any planned programmatic or scholarly outcomes that had resulted from the VTT. The authors evaluated changes in attendee perceptions resulting from the VTT session using reflective pre−/post- questions on the post-session survey. Changes in attendees' awareness of and perceptions regarding organizational resources available to them were also assessed through seven items on the CLIP follow-up survey. Demographic data were also collected. Although it was not clear at the time of the VTT when it could be possible to meet in-person in large groups again, a plan was made to repeat the VTT in a future live session and compare outcomes of the two different formats.

## 3 Results

Seven groups of approximately 10 individuals, representing 55 unique institutions, comprised the CLIP groups. Thirty-eight of the 70 VTT attendees who were invited and eligible to complete the follow-up survey successfully completed it (response rate = 54%). Of the respondents, 73.7% held administrative roles within the field of pharmacy academia and 60.5% reported participation in at least one formal leadership training program (Table 1). Approximately 29% of the respondents were in academia <10 years and 24% had been in academia ≥20 years.

**Table 1:** Respondents' professional demographics.

Demographic Variable	Values	Quantities n (%)
Academic Rank	Assistant Professor	9 (23.7%)
	Associate Professor	13 (34.2%)
	Full Professor	14 (36.8%)
	Other (Graduate Student)	2 (5.3%)
# Years in Academia	0–4	4 (10.5%)
	5–9	7 (18.4%)
	10–14	10 (26.3%)
	15–19	7 (18.4%)
	20–24	6 (15.8%)
	25–29	1 (2.6%)
	≥ 30	2 (5.3%)
	No response	1 (2.6%)
Administrative Role within Pharmacy Education	Dean	1 (2.6%)
	Assistant/Associate Dean	8 (21.1%)
	Department Chair	6 (15.8%)
	Assistant/Associate/Vice Department Chair	2 (5.3%)
	Director	3 (7.9%)
	Associate/Assistant Director	1 (2.6%)
	Unspecified/Other administrative position	5 (13.2%)
	Committee Chair	2 (5.3%)
	No administrative position	10 (26.3%)
Prior Leadership Training⁎	American Association of Colleges of Pharmacy Academic Leadership Fellows Program (ALFP)	8 (21.1%)
	American College of Clinical Pharmacy (ACCP) Leadership & Management Academy	4 (10.5%)
	International Pharmaceutical Federation (FIP) Leadership training program	0 (0%)
	Other Leadership Training Program	14 (36.8%)
	No Leadership Training Program	15 (39.5%)

⁎ 2 respondents selected 2 Prior Leadership Training Programs.

As shown in Table 2, following the VTT there was a significant increase in considering the LD SIG helpful for networking, leadership-related scholarship, teaching, and service as well as likelihood to collaborate with LD SIG members (*p* < 0.05 for all outcomes). Attendees also reported increased familiarity with (*p* = 0.0215) and willingness to utilize (*p* = 0.0018) LD SIG organizational resources (website, podcast, toolkit, etc). Other key quantitative findings included 18.4% of attendees continuing communications with CLIP groups post-VTT and 13.2% of respondents indicating successful implementation of CLIP ideas post-VTT (Table 3). Table 3 also includes descriptions of the CLIP ideas that were successfully implemented. Qualitative findings included one-third of respondents indicating that the VTT inspired other novel leadership initiatives (differing from CLIP ideas), including programmatic development (leadership podcast series for students, virtual coffee hour, webinars), scholarship collaboration (Scholarship of Teaching and Learning grant, LD SIG Award/Rubric) and greater organizational involvement from participants.

**Table 2:** Quantitative outcomes.

Survey Items:	Mean Score		
	Pre-CLIPs	Post-CLIP	*p*-value
I consider the LD SIG a helpful group for networking to help inform my leadership related program initiatives	3.64	3.92	0.0300
I consider the LD SIG a helpful resource for collaboration for leadership-related scholarship	3.46	3.90	0.0046
I consider the LD SIG a helpful resource for collaboration for leadership-related teaching	3.56	3.90	0.0078
I consider the LD SIG a helpful resource for leadership-related service	3.49	3.77	0.0410
I have familiarity with the LD SIG resources available to me online (e.g. LDPEcast Leadership Development podcast, Leadership Toolkit, LD SIG Book Blog, etc.)	3.49	4.00	0.0107
I am likely to use the LD SIG resources available to me online (e.g. LDPEcast Leadership Development podcast, Leadership Toolkit, LD SIG Book Blog, etc.) when planning leadership initiatives	3.28	3.87	0.0006
I am likely to reach out to a member of the LD SIG regarding a leadership-related topic or query	3.36	3.74	0.0024

**Table 3:** Qualitative outcomes.

Type of Outcomes:	Specific Examples of Outcomes Planned:
Scholarly Outcomes	• Development of a new LD SIG Award/Rubric • Submission of an AACP Scholarship of Teaching and Learning (SOTL) grant application
Programmatic Outcomes	• New National and Global Webinars on Leadership Development • Development of a new leadership program/leadership toolkit at an institution
Additional Outcomes	• New partnerships across universities built around shared leadership development interests • Continued use of the Virtual Structured Introduction Forms including the creation of a new “LD SIG Networking Database” available to all members
New Ideas Generated from CLIP Groups	• Alignment of tenure and promotion criteria to incentivize entrepreneurship and innovation • Incorporation of leadership projects into APPEs • Development of a student entrepreneurship track • Creation of innovative co-curricular programs with faculty from a variety of institutions presenting leadership content • Development of a virtual Leadership Curriculum for mid-career pharmacist faculty • Development of a co-curricular leadership development podcast series • Creation of a leadership mentoring program.

Of the 31 respondents indicating that they did not communicate with their CLIP groups following the VTT, 64.5% (*n* = 20) responded that the rationale for no further group communication was failure to establish a path forward during the initial meeting. The next most common rationale was attendees being unsure of how to proceed following the VTT 29.0% (*n* = 9). Scheduling conflicts with other CLIP group members 3.2% (*n* = 1) and lack of interest 9.7% (*n* = 3) did not appear to be common rationales for the lack of communication occurring within some CLIP groups following the VTT. Other rationales for poor communication following the VTT included health concerns, the COVID-19 pandemic, and family emergencies. Attendees were also asked to identify any barriers to follow-up communication with their CLIP groups encountered following the VTT. The two most frequently encountered barriers were insufficient traction of the initial idea and lack of time (41.9% (*n* = 13) for both). Other barriers included lack of alignment with work priorities (12.9%, *n* = 4). Barriers to successful implementation of the CLIP ideas generated during the VTT included discovery that the CLIP idea generated had already been researched with forthcoming publications, lack of establishment of accountability for follow-up during the CLIP group meeting time, competing responsibilities and priorities, failure to designate a leader to carry the CLIP idea forward, lack of time to fully develop an idea during the VTT, and lack of follow-up communication between CLIP group members.

## 4 Discussion

Healthcare leaders and managers face an ever-evolving field. Health system strengthening relies on management's ability to adapt to challenges.^11^ The VTT helped organizational leadership respond to an unprecedented challenge in the global COVID-19 pandemic while fostering collaboration among colleagues. Almost 50% of post-VTT survey respondents were mid-career individuals with some type of formal training in the VTT content area (i.e. leadership development) and most respondents (approximately 70%) had relevant leadership/administrative experience. Thus, our findings demonstrate that other organizations could benefit from VTTs that are directed toward a mid-career audience with some level of formal training and leadership experience in the relevant topic area. We anticipate that the utility of VTTs could extend beyond leadership development-related topics into other realms of organizational development to help facilitate collaboration. Given the generally positive responses from survey respondents, VTTs represent a great opportunity to provide value to a variety of organizations. Besides providing insight into the ideal organizational constituents benefitting from a VTT, our study also informs VTT design components that might contribute to successful implementation of the ideas generated. Organizations should allocate enough time for VTT attendees to brainstorm enough to form a clear idea and vet its validity or novelty. It also appears important to develop a specific and intentional path forward for future communication among group members, based on our respondents' feedback. While group members may be interested in continued implementation work following the VTT, a concrete follow-up communication plan that can be easily prioritized despite busy schedules appears to be essential, including a designated leader to facilitate communication and hold the group accountable to implementation and continued follow-up until the desired deliverables have been produced. While a few of our CLIP groups were able to produce tangible programmatic and scholarly deliverables as VTT outcomes, the other groups reported being most likely to succumb to failure when these elements were not present.

Thus, the outcomes of our study provide previously unpublished insights into the demographic, group dynamics, and design of a VTT that is associated with statistically significant increases in positive perceptions toward the organization hosting the VTT regarding networking, scholarly collaboration, educational collaboration, and professional service opportunities. In addition, we also noted significant improvements in attitudes regarding organizational engagement in our study findings.

The use of reflective pre−/post- survey items within our VTT Outcomes assessment questionnaire is a strength of our study in avoiding response-shift bias. Response-shift bias can occur within paired datasets in which respondents are asked to rate themselves on a Likert scale before and after an intervention is provided, and can occur if the intervention changes the standard by which the study subject evaluates him or herself.^12^ While a response rate of 54% to an electronically distributed survey instrument is reasonable, our small sample size represents an obvious limitation of the study. Other limitations include the possibility of introducing recall bias into the study sample by surveying attendees 6 months after the event, but this delay was also necessary to allow sufficient time for CLIP implementation following the VTT.

The organization hosting the VTT has also directly benefited from the session. On the Virtual Structured Introduction Form used to prepare for the VTT, contact information along with primary leadership interest areas, institutional leadership needs and institutional strengths related to leadership development were gathered from approximately 100 individuals prior to the session. This information has now been compiled and repurposed to create a new LD SIG Networking Database. The VTT has provided enduring benefits for both session attendees and LD SIG members for years to come. Additionally, attendees subsequently created a new LD SIG Innovations in Leadership Award & Rubric for candidate selection following the VTT.

## 5 Conclusion

A VTT led to development and implementation of CLIPs, birthed novel leadership programs and scholarly initiatives, and fostered partnerships across universities built around shared leadership development interests. In addition to these benefits for attendees, the hosting organization realized several benefits as well. For organizations seeking outcomes similar to those observed for this VTT, such as significant improvements in considering the organization helpful for networking and collaboration and increasing familiarity and willingness to use organizational resources, the professional demographics of our respondents provide insight into which organizational constituents might similarly benefit. Thus, organizations could consider targeting an audience that is mid-career, with some level of formal training and leadership experience in the relevant topic area for VTT participation.

## CRediT authorship contribution statement

**Whitney D. Maxwell:** Writing – review & editing, Writing – original draft, Project administration, Methodology, Investigation, Formal analysis, Data curation, Conceptualization. **Kerry K. Fierke:** Writing – review & editing, Writing – original draft, Project administration, Methodology, Conceptualization. **Gregory M. Zumach:** Writing – review & editing, Writing – original draft, Validation, Methodology, Investigation, Conceptualization.

## Declaration of competing interest

None.

## Appendix A Supplementary data

Supplementary material: Post-session follow-up survey instrument Image 1

## References

[1] Harasim L. (2007). Assessing online collaborative learning: a theory, methodology, and toolset. Flexible Learn Inf Soc.

[2] Harasim L. (2017).

[3] Crocetti G. (2015).

[4] Ramirez-Zamora A., Giordano J., Gunduz A. (2020). Proceedings of the seventh annual deep brain stimulation think tank: advances in neurophysiology, adaptive DBS, virtual reality, Neuroethics and technology. Front Hum Neurosci.

[5] Beatty A.L., Brown T.M., Corbett M. (2021). Million hearts cardiac rehabilitation think tank: accelerating new care models. Circ Cardiovasc Qual Outcomes.

[6] Lewinski A.A., Sullivan C., Allen K.D., Crowley M.J. (2021). Accelerating implementation of virtual Care in an Integrated Health Care System: future research and operations priorities. J Gen Intern Med.

[7] Mathioudakis A.G., Custovic A., Deschildre A., Ducharme F.M. (2020). Research priorities in pediatric asthma: results of a global survey of multiple stakeholder groups by the pediatric asthma in real life (PeARL) think tank. J Allergy Clin Immunol Pract.

[8] Rahman R., Ventz S., McDunn J., Louv B. (2021). Leveraging external data in the design and analysis of clinical trials in neuro-oncology. Lancet Oncol.

[9] Vedam-Mai V., Deisseroth K., Giordano J., Lazaro-Munoz G., Chiong W. (2021). Proceedings of the eighth annual deep brain stimulation think tank: advances in optogenetics, ethical issues affecting DBS research, neuromodulatory approaches for depression, adaptive neurostimulation, and emerging DBS technologies. Front Hum Neurosci.

[10] Maxwell W.D., Fierke K.K., Zumach G.M. (2022). A Virtual “Think Tank” to Establish Collaborative Leadership Initiative Plans (“CLIPs”). INNOVATIONS in Pharmacy.

[11] Ginter P.M., Duncan W.J., Swayne L.E. (2018). Strategic Management of Health Care Organizations.

[12] Howard G.S. (1980). Response-shift Bias: a problem in evaluating interventions with pre/post self-reports. Eval Rev.

