# Supplementary material for: Outcomes of a “virtual think tank” to establish collaborative leadership initiative plans (“CLIPs”)
Source: Explor Res Clin Soc Pharm. 2024 Jan 18;13:100409. doi: 10.1016/j.rcsop.2024.100409 (PMC10839148; doi:10.1016/j.rcsop.2024.100409)
Supplement: Supplementary file 1 — Supplementary material: Post-session follow-up survey instrument [file mmc1.docx]

Appendix A. Post-session follow-up survey instrument

Please use these definitions to assist you with completing the following assessment of the July 2020 AACP Virtual Annual Meeting Leadership Development Special Interest Group (LD SIG) Live Networking Zoom session on July 16, 2020:

Collaborative Leadership Initiative Plan ("CLIP") group - The small group of individuals who you met with in a virtual Zoom breakout room during the LD SIG Live Networking Session

CLIP idea - The ideas for programmatic development, scholarly collaboration, etc. that were discussed in your virtual small group (CLIP group) meeting during the LD SIG Live Networking Session

CLIP group work - The discussions that occurred in the virtual small group (CLIP group) interactions during the LD SIG Live Networking Session

At the July 2020 Virtual AACP Annual Meeting last summer, which CLIP group did you participate in during the LD SIG live networking Zoom session?

- Curricular Leadership Program Development
- Co-Curricular Leadership Program Development
- Faculty Development Program Creation
- Leadership, Entrepreneurship, & Innovation Group A
- Leadership, Entrepreneurship & Innovation Group B
- Leadership in Experiential Education
- Leadership-related Assessment Tools

Have you communicated in any way with your CLIP group since the AACP Virtual Annual Meeting that occurred in July 2020?

- Yes
- No

What was the rationale for no further communication with your CLIP group?

I wasn't sure how to proceed after the Meeting

Scheduling conflicts with CLIP group members

We didn't establish a path forward during the initial meeting

I was not interested in pursuing further

Other

Please specify "Other"

What were the barriers to follow-up communication with your CLIP group?

Insufficient traction of initial idea

Lack of personal interest in initial idea

Lack of time to pursue initial idea

Idea did not align with priorities

Other

Please specify "Other"

Are you still actively communicating with your CLIP Yes

group? No


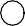

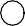


Which of the following best describes the follow-up Minimal email follow-up (< 2 email exchanges) communications occurring with your CLIP group since Occasional email follow-up (2-5 email exchanges) the AACP Virtual Annual Meeting? Frequent email follow-up (>5 email exchanges)


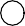

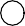

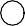

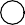

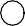

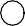

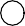


Minimal virtual follow-up (< 2 virtual meetings) Occasional virtual follow-up (2-5 virtual meetings) Frequent virtual follow-up (>5 virtual meetings)

Other

Please specify "Other"

What is the longest timeframe that you and any newly < 1 month acquainted colleague from your CLIP group have 2-3 months

remained in communication? 4-6 months


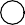

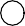

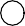

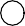


We are still in communication

Are you aware of your CLIP idea being implemented or Yes


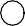

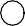


further developed in any way at your or others' No

institution after the AACP Virtual Annual Meeting?

What were the barriers to successful implementation or further development of your CLIP idea?

Which of the following LD SIG resources helped facilitate implementation or further development of your CLIP idea? (select all that apply)

AACP Connect Community posts to identify additional ideas/collaborators

LD SIG Leadership Toolkit Virtual Symposium Recordings

LEaDeR Manuscript category availability in CPTL or Innovations in Pharmacy

LD SIG Book Blog postings

LD SIG Journal Clubs or LD SIG Book Clubs

LD SIG Webinar topics/slides/recordings

AACP Annual Meeting Session topic archives

LD SIG Leadership Development in Pharmacy Education (LDPE) Podcast

LEADERx Newsletter or LD SIG Digital Magazine archives

LD SIG Poster or Manuscript of the Year archives

Other

None

Please specify "Other"

Are there any EXISTING programmatic (e.x. new course, new co-curriculum, etc.) OR scholarly outcomes of

your CLIP group work (e.x. new research Yes


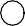

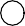


collaboration, new manuscript in progress, etc.)? No

What are the EXISTING PROGRAMMATIC outcomes of your CLIP group work?

What are the EXISTING SCHOLARLY outcomes of your CLIP group work?

Are there any PLANNED PROGRAMMATIC (e.x. new course, new co-curriculum, etc.) OR SCHOLARLY outcomes of your CLIP group work (e.x. new research Yes


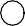

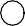


collaboration, new manuscript in progress, etc.)? No

What are the PLANNED PROGRAMMATIC outcomes of your CLIP group work?

What are the PLANNED SCHOLARLY outcomes of your CLIP group work?

Did the CLIP group work inspire or generate other Yes

unique leadership ideas or initiatives? No


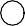

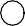


Please explain

Did the CLIP group work inspire or generate other Yes

unique leadership networking or communications? No


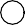

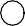


Please explain

**I consider the LD SIG a helpful group for networking to help inform my leadership-related**

**program initiatives**

|  | **Strongly**  **disagree** | **Disagree** | **Neutral** | **Agree** | **Strongly**  **Agree** |
| --- | --- | --- | --- | --- | --- |
| BEFORE CLIP groups met at 2020 AACP Virtual Annual Meeting |  |  |  |  |  |
| AFTER CLIP groups met at 2020 AACP Virtual Annual Meeting |  |  |  |  |  |

|  | **Strongly**  **disagree** | **Disagree** | **Neutral** | **Agree** | **Strongly**  **Agree** |
| --- | --- | --- | --- | --- | --- |
| BEFORE CLIP groups met at 2020 AACP Virtual Annual Meeting |  |  |  |  |  |
| AFTER CLIP groups met at 2020 AACP Virtual Annual Meeting |  |  |  |  |  |

**I consider the LD SIG a helpful resource for collaboration for leadership-related scholarship****rship-related scholarship**

|  | **Strongly**  **disagree** | **Disagree** | **Neutral** | **Agree** | **Strongly**  **Agree** |
| --- | --- | --- | --- | --- | --- |
| BEFORE CLIP groups met at 2020 AACP Virtual Annual Meeting |  |  |  |  |  |
| AFTER CLIP groups met at 2020 AACP Virtual Annual Meeting |  |  |  |  |  |

|  | **Strongly**  **disagree** | **Disagree** | **Neutral** | **Agree** | **Strongly**  **Agree** |
| --- | --- | --- | --- | --- | --- |
| BEFORE CLIP groups met at 2020 AACP Virtual Annual Meeting |  |  |  |  |  |
| AFTER CLIP groups met at 2020 AACP Virtual Annual Meeting |  |  |  |  |  |


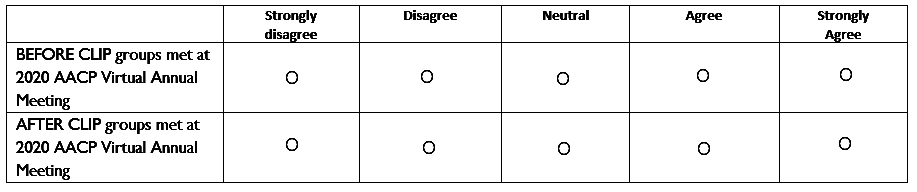


**I consider the LD SIG a helpful resource for collaboration for leadership-related teaching**


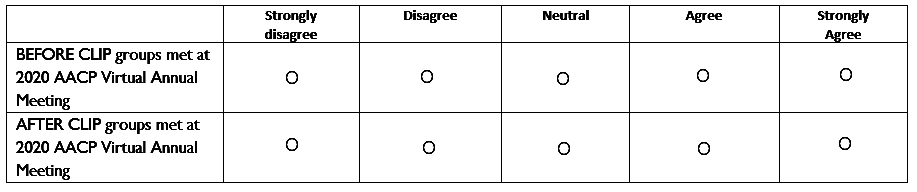


**I consider the LD SIG a helpful resource for leadership-related service**


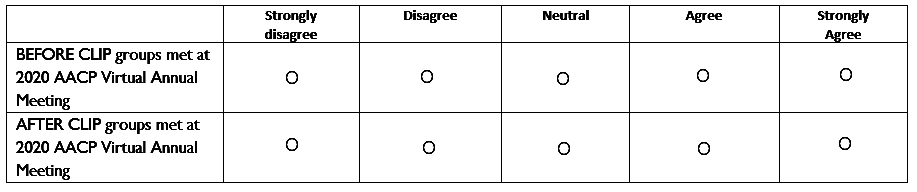


**I have familiarity with the LD SIG resources available to me online (e.g. LDPEcast Leadership**

**Development podcast, Leadership Toolkit, LD SIG Book Blog, etc.)**


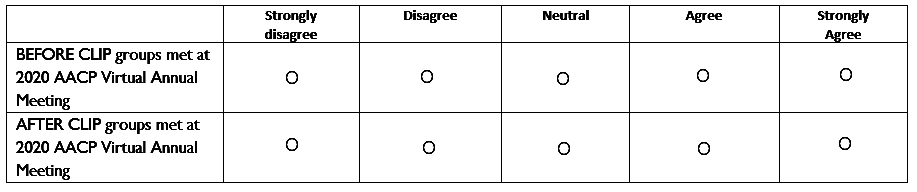


**I am likely to use the LD SIG resources available to me online (e.g. LDPEcast Leadership**

**Development podcast, Leadership Toolkit, LD SIG Book Blog, etc.) when planning leadership initiatives**


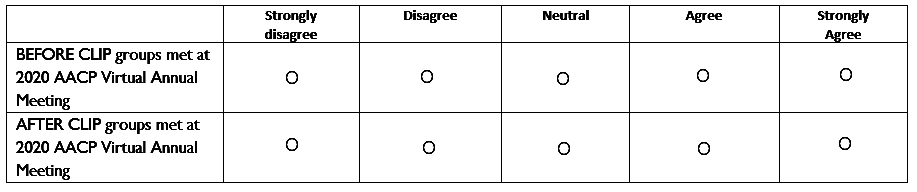


**I am likely to reach out to a member of the LD SIG regarding a leadership-related topic or**

**query**


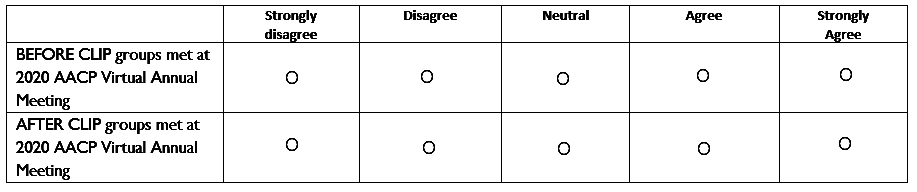


In your own words, please describe your general experience with you CLIP group during the virtual Annual Meeting:

_________________________________________________________________________________________

Please provide your Academic Rank Full professor Associate professor Assistant professor Instructor


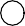

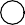

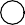

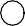

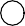


Other

Please specify "Other"

Please provide the number of years you have been in 0-4

academia 5-9


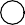

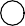

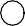

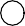

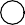

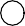

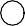


10-14

15-19

20-24

25-29

30+

Please describe your role within pharmacy education at your institution (e.x. administrative titles, etc.)

Please describe your prior leadership training (select all that apply)

Academic Leadership Fellows Program (ALFP)

ACCP Leadership & Management Academy

FIP Leadership training program

Other formal leadership training/course

None

Please specify “Other formal leadership training/course”: ________________________

\
